# Lansoprazole as a rescue agent in chemoresistant tumors: a phase I/II study in companion animals with spontaneously occurring tumors

**DOI:** 10.1186/1479-5876-9-221

**Published:** 2011-12-28

**Authors:** Enrico P Spugnini, Alfonso Baldi, Sabrina Buglioni, Francesca Carocci, Giulia Milesi de Bazzichini, Gianluca Betti, Ilaria Pantaleo, Francesco Menicagli, Gennaro Citro, Stefano Fais

**Affiliations:** 1 SAFU Department, Regina Elena Cancer Institute, Rome, Italy; 2 Department of Biochemistry, Second University of Naples, Naples, Italy; 3 Ambulatorio Veterinario "Le Accademie", Rome, Italy; 4 Ambulatorio Veterinario "Farnesina", Rome, Italy; 5 Centro Veterinario Gianicolense, Rome, Italy; 6 Anti-Tumor Drug Section, Dept. of Drug Research and Medicine Evaluation, National Institute of Health (ISS), Rome, Italy

**Keywords:** chemotherapy, lansoprazole, mitoxantrone, carboplatin, proton pump

## Abstract

**Background:**

The treatment of human cancer has been seriously hampered for decades by resistance to chemotherapeutic drugs. Mechanisms underlying this resistance are far from being entirely known. A very efficient mechanism of tumor resistance to drugs is related to the modification of tumour microenvironment through changes in the extracellular and intracellular pH. The acidification of tumor microenvironment depends on proton pumps that actively pump protons outside the cells, mostly to avoid intracellular acidification. In fact, we have shown in pre-clinical settings as pre-treatment with proton-pumps inhibitors (PPI) increase tumor cell and tumor responsiveness to chemotherapeutics. In this study pet with spontaneously occurring cancer proven refractory to conventional chemotherapy have been recruited in a compassionate study.

**Methods:**

Thirty-four companion animals (27 dogs and 7 cats) were treated adding to their chemotherapy protocols the pump inhibitor lansoprazole at high dose, as suggested by pre-clinical experiments. Their responses have been compared to those of seventeen pets (10 dogs and 7 cats) whose owners did not pursue any other therapy than continuing the currently ongoing chemotherapy protocols.

**Results:**

The drug was overall well tolerated, with only four dogs experiencing side effects due to gastric hypochlorhydria consisting with vomiting and or diarrhea. In terms of overall response twenty-three pets out of 34 had partial or complete responses (67.6%) the remaining patients experienced no response or progressive disease however most owners reported improved quality of life in most of the non responders. On the other hand, only three animals in the control group (17%) experienced short lived partial responses (1-3 months duration) while all the others died of progressive disease within two months.

**Conclusions:**

high dose proton pump inhibitors have been shown to induce reversal of tumor chemoresistance as well as improvement of the quality of life in pets with down staged cancer and in the majority of the treated animals PPI were well tolerated. Further studies are warranted to assess the efficacy of this strategy in patients with advanced cancers in companion animals as well as in humans.

## Introduction

Cancer initiation, progression, and invasion occur in a complex and dynamic microenvironment which depends on the hosts and sites where tumors develop. The response to chemotherapy by tumor cells depends on the concentration of cytostatics accumulated within the cells. The accumulation of anticancer drugs in tumor cells is dependent on functional expression of efflux transporters, but also on the pH of extracellular microenvironment. However, while the role of chemotransporters in the chemoresistance of malignant tumors has been very well documented, little is known about the role of tumor acidity and mechanisms underlying tumor acidification, including proton exchangers and their impact on the chemosensitivity of cancer cells. Tumor cells rely on H^+ ^exchangers to relieve themselves from the dangerous protons byproduct of cancer metabolism that could trigger a cascade of lytic enzymes that ultimately would lead to self-digestion. Among these the most prominent are the vacuolar H^+^-ATPases (V-ATPases). V-ATPases are ATP dependent H^+ ^transporters that utilize the energy freed by the hydrolysis of ATP with the active transport of protons from the cytoplasm to the lumen of intracellular compartments or, if located within the cytoplasmic membrane, the extracellular compartment [1-4]. Two important physiological mechanisms of regulating V-ATPase activity *in vivo *are reversible dissociation of the domain carrying ATP from the proton exchanger domain and changes in coupling efficiency of proton transport and ATP hydrolysis [5-12]. Malignant tumor cells overexpress lysosomal proteins on the cell surface, with abnormal lysosomal activities, possibly involving deranged V-ATPase function [13,14]. The acidic tumor environment is a consequence of anaerobic glucose metabolism resulting in accumulation of acid byproducts such as lactates. This involves the upregulation of hypoxia-inducible factor 1α [15] or can be dependent on inadequate tumor perfusion, hypoxia secondary to disordered tumor growth or enhanced transmembrane pH regulation [16]. These pumps, coupled with other ion exchangers, play a paramount role in the establishment and maintenance of malignant tumor microenvironment and their action lead to the selection of more aggressive cell phenotypes able to survive in this highly hostile microenvironment.

V-ATPases play a critical role in the maintenance of an appropriate relatively neutral intracellular pH, and an acidic extracellular pH by actively excreting protons either through ion exchange mechanisms or by segregating H^+ ^within cytoplasmic organelles that are subsequently expelled [17]. It is hypothesized that the low extracellular pH of tumors might trigger proteases (MMP-2, MMP-9, cathepsin B, and cathepsin L), leading to the dissolution of extracellular matrix. Proton exchangers-mediated acidification of tumor microenvironment significantly contributes to tumor invasion and dissemination [18,19]. In fact, it has been shown that by inhibiting V-ATPases through RNA interference, it was possible to prevent cancer metastases in a murine model [19]. This could be a novel strategy to deal with the process of tumor dissemination through the increase of the extracellular tumor pH, thus inhibiting the activation of tumor proteases. From the therapeutic point of view, the changes in the pH gradient occurring between the intracellular and the extracellular compartments as well as the pH gradient between the cytoplasm and the intracellular organelles can be significantly involved in the mechanism of drug resistance [20-22]. There are several proposed mechanisms involved in this phenomenon, including decreased uptake or neutralization of weakly basic drugs by the acidic tumor microenvironment or the confinement of chemotherapy drugs within lysosomal vesicles [21-25]. An accelerated turnover of acidic vesicles may represent an additional tumor strategy of drug resistance based on counteracting current transportation [26,27]. Interestingly, the expression of proton pumps is increased in chemoresistant phenotypes and is increased by anticancer drugs [28-31]. Investigation in xenograft models with different human tumor histologies have shown as proton pump inhibition may on one hand induce chemosensibilization, on the other hand trigger a clear tumor cytotoxicity [26,27]. Proton pump inhibitors are normally adopted in the treatment of gastritis, Zollinger-Ellison syndrome and, limitedly to veterinary oncology, gastric hyperacidity secondary to mast cell tumors in dogs and cats [32-36]. These drugs have been shown to be highly effective at inhibiting V-ATPases in vitro and well tolerated and extremely efficacious in murine models, resulting in increased chemotherapy efficacy and improved tumor control [27,31,37,38]. Moreover, according to data reported in current veterinary literature, at least in two dogs with gastrinoma, PPI therapy with omeprazole resulted in survivals in excess of 2 years. Such long survivals, are potentially more due to tumor control secondary to cancer microenvironment manipulation than to palliation of hyper acidic syndrome and prevention of gastrointestinal ulcerations [33,35]. Aim of this study was to investigate the feasibility, tolerability and efficacy of high dose proton pump inhibitor lansoprazole as a rescue to revert chemoresistance in companion animals affected by neoplasms non responsive to anticancer drugs.

## Methods

### Patient selection

Privately owned canine and feline patients with advanced neoplasms that showed progression despite chemotherapy were selected for the study. Upon tumor escape from pharmaceutical control, owners were offered three options: a) discontinuation of all therapies, b) continuation of chemotherapy alone, c) continuation of chemotherapy with the addition of high dose lansoprazole.

Previous informed consent was obtained from the owners. In order to be enrolled in the study, according to the Italian law (116/92) and the guidelines defined by the ethical committee of the National Cancer Institute " Regina Elena" of Rome, Italy, patients, staged according to the World Health Organization (WHO) grading system, were considered eligible if they fulfilled the following criteria:

1 Normal renal function (normal serum blood urea nitrogen [BUN], creatinine, phosphorus, and urine specific gravity).

2 Absence of underlying life threatening diseases or other medical complications (e.g. diabetes mellitus).

3 Compliance of the owner for follow-up rechecks.

4 A presumptive life expectancy of at least four weeks.

5 Overall performance status assessed according to the modified Karnowsky system, had to be less than 3 (Table 1).

**Table 1:** Modified Kamofsky's performance criteria

Grade	Criteria
0	Fully active, performs at predisease level

1	Activity less than predisease level; able to function as acceptable pet

2	Severely compromised activity; ambulatory only to point of eating, sleeping, and consistently eliminating in acceptable areas.

3	Completely disabled; must be force fed; unable to defecate or urinate in acceptable areas

4	Dead

Staging process included a thorough anamnesis, physical examination, caliper or ultrasonographic measurement of the neoplasm, complete blood cell count (CBC), serum biochemistry profile, thoracic radiographs (three projections: two laterals and one ventro-dorsal), and abdominal ultrasonography. In order to confirm the diagnoses, histological re-examination of the biopsies were performed following standard protocols, using Hematoxylin/Eosin and Hematoxylin/Van Gieson stainings by one of the authors (AB).

#### Treatment

Twenty-seven privately owned dogs and seven cats presented to the Regina Elena Cancer Institute with clinically chemoresistant neoplasms and were entered the proton pump inhibitors arm in the modified phase I/II study between September 2009 and April 2011. Similarly, a group of seventeen pets whose owners declined to enroll their pets in the study but chose to continue standard chemotherapy was followed as control group.

Treatment protocol in the experimental arm consisted with lansoprazolo at 5 mg/Kg SID for three consecutive days, at the time of each chemotherapy administration, to decrease tumor pH and increase response to therapy, followed by four days at the dose of 1 mg/Kg SID to prevent gastric hyperacidity rebound. This schedule has been chosen on the basis of previous studies on rodents and based on the currently ongoing clinical trials in humans [27].

Response to treatment in terms of toxicity and tumor response were assessed prior each therapy. At that time a physical exam and tumor measure were performed. Moreover thoracic radiographs and abdominal ultrasonography were performed every two months to check for tumor spread. Toxicity was defined as disease processes that occurred secondary to therapy and accordingly scored (table 2). In order to have the best assessment of therapy toxicoses, after every therapy owners were sent home with a questionnaire to be completed in order to record possible gastrointestinal side effects of the protocol (Table 3). Tumor response was defined as follows:

**Table 2:** Modified Eastern Cooperative Oncology Group evaluation

Toxicity/Grade Signs	Duration
Hospitalization	Days

0	0

1	1

2	2-3

3	4-5

4	≥ 5

Neutropenia	

0	≤ 500 neutrophils/mL

1	1,500-2,500 neutrophils/mL

2	≥ 2,500 neutrophils/mL

3	500-999 neutrophils/mL

4	1,000-1,499 neutrophils/mL

Anorexia	

0	None

1	Inappetance

2	Anorexia ≤ 3 days duration

3	Anorexia > 3 days but < 5 days

4	Anorexia ≥ 5 days 10% weight loss

Vomiting	

0	None

1	Nausea

2	Sporadic, self-limiting

3	1-5 episodes per day, < 2 days

4	6-10 episodes per day, hospitalized

Diarrhea	

0	None

1	Soft stools, responds to dietary modification

2	1-4 watery stools per day, < 2 days

3	4-7 watery stools per day or > 2 days

4	> 7 watery stools per day or bloody, hospitalized

Infection	

0	None

1	No medication

2	Required medication

3	Debilitating

4	Threatening

**Table 3:** Daily evaluation form sent home and made out by the owners

Vomiting				
None	3 episodes per day	5 episodes per day	> 5 episodes per day	> 5 per day OR
	OR	OR	OR	days lasting > 4 days and
	vomiting lasting 2 days	vomiting lasting 4 days	for > 4 days	life threatening
**Diarrhea**				

None	2 more stools	6 more stools	> 6 more stools	> 6 and life
	than normal	than normal	than normal	hospitalized
**Nausea**				

None	Appetite loss with	Salivating or lip	Salivation or lip	Salivation/ lip
	normal eating habits	smacking for 12 hrs	smacking for 24 hrs	smacking > 24 h
**Appetite**				

Normal	With treats or diet	Appetite loss for 3 days OR	Appetite loss for 5 days OR	Loss > 5 days
	change, ate 100%	With treats or diet	With treats or diet	OR
		change, ate 50% of normal	change, ate few bites	No interest,
				no appetite
**Flatulence**				

Normal	1-2 episodes per day	2-4 episodes per day	4-6 episodes per day	> 6 episodes per day
**Activity**				

Normal	Mild lethargy	Moderate lethargy, difficulty	Severe lethargy, only	Unable to
		with daily activities	gets up to go outside	rise on own

**Complete Remission **(CR) - the disappearance of all evidence of cancer in all sites for a defined period of time.

**Partial Remission **(PR) - the decrease in size of all tumors by 50% or greater as measured by the sum of the product of two diameters of each tumor for a defined period of time.

**Stable Disease **(SD) - the decrease of < 50% or an increase of < 25% in the sum of the product of two diameters for a defined period of time.

**Progressive Disease **(PD) - the increase of 25% or more in the sum of the product of two diameters for a defined period of time.

**No evidence of disease **- absence of tumor growth (local recurrence or distant metastases) after PPI and chemotherapy for a defined period of time.

Finally, the owners were questioned prior to each therapy on the activity level, performance status and food and water consumption of their animals. Treatment were scheduled to be continued for six additional months in lymphoma patients, upon achievement of complete remission and for 3 additional months in patients with solid tumors.

## Results

### Dogs

PPI COHORT: Twenty-seven dogs entered the study over a 19 months period and their characteristics and treatment protocols are summarized in tables 4, 5 and 6. There were 8 mixed breed dogs, 4 Boxers, 3 West Highland White Terriers, and one each of the following breeds: Great Dane, Bull Mastiff, Bull dog, Schnauzer, Husky, Labrador, Rottweiler, German Shepherd, Argentine Dogo, Setter, Poodle, and Beagle. There were 16 males and 11 females (all of them spayed). The age ranged from 5 to 15 years with a mean a mode of 10 years. There were eleven cases of lymphoma, three of osteosarcoma, three of mammary carcinoma, two of bladder carcinoma, and finally, one each of the following: acute lymphocytic leukemia, hemangiosarcoma, anal sac carcinoma, melanoma, fibrosarcoma, oral squamous cell carcinoma, nasal carcinoma, mammary carcinosarcoma. All the patients had previous treatment with chemotherapy: lymphoma patients had been previously treated with first and rescue protocols (Madison Wisconsin or COP and upon failure, rescue with MOPP. Protocols details are provided in tables 4 and 5). Upon failure they were treated with reinstituted MOPP coupled with PPI. Only exception a patient with cutaneous lymphoma who developed intolerance to vincristine and, in consideration of its reduced cardiac fraction shortening, was treated with the doxorubin analogue mitoxantrone, coupled with PPI. Dogs with solid tumors who had been treated with a variable number of cycles of platinum drugs and/or anthracyclines (or their synthetic analogue mitoxantrone), were treated, upon tumor progression, with mitoxantrone in association with PPI. The only exceptions were a patient affected by vesical transitional cell carcinoma whose owners elected to be treated with piroxicam alone, because of financial issues, and three dogs with osteosarcoma whose tumor type is not responsive to veterinary adopted chemotherapy regimens that were treated with the calcifying agent clodronate. Upon failure, clodronate has been reinstituted with high dose PPI. In table 6 are summarized the data on the PPI cohort.

**Table 4:** Madison Wisconsin lymphoma protocol

Week 1:		Vincristine 0.7 mg/m^2 ^IV
		Asparaginase 400 IU/kg IM
		Pre-med with chlorphenamine: small-medium 2.5-
		5 mg IM; medium-large 5-10 mg IM
		Prednisolone 2 mg/kg PO SID
	Start antacids:	zantac 2 mg/kg PO BID
		antepsin < 20 kg 500 mg, > 20 kg 1-2 g PO TID
**Week 2:**		Cyclophosphamide 250 mg/m^2 ^PO or IV (+ NaCl)
		Prednisolone 1.5 mg/kg PO SID
	**Stop antacids:**	after week 2

**Week 3:**		Vincristine 0.7 mg/m^2 ^IV
		Prednisolone 1 mg/kg PO SID

**Week 4:**		Doxorubicin 30 mg/m^2 ^IV (+ 0.9% NaCl)
		Pre-med with chlorphenamine and
		metoclopramide 0.5-1 mg/kg IM/SC)
		Prednisolone 0.5 mg/kg PO SID

**Week 6:**		Vincristine 0.7 mg/m^2 ^IV

**Week 7:**		Cyclophosphamide 250 mg/m^2 ^PO or IV (+ NaCl)

**Week 8:**		Vincristine 0.7 mg/m^2 ^IV

**Week 9:**		Doxorubicin 30 mg/m^2 ^IV (+ 0.9% NaCl) Pre-med with chlorphenamine + metoclopramide

**Week 11:**		Vincristine 0.7 mg/m^2 ^IV

**Week 13:**		Cyclophosphamide 250 mg/m^2 ^PO or IV (+ NaCl)

**Week 15:**		Vincristine 0.7 mg/m^2 ^IV

**Week 17:**		Doxorubicin 30 mg/m^2 ^IV (+ 0.9% NaCl) Pre-med with chlorphenamine + metoclopramide

**Week 19:**		Vincristine 0.7 mg/m^2 ^IV

**Week 21:**		Cyclophosphamide 250 mg/m^2 ^PO or IV (+ NaCl)

**Week 23:**		Vincristine 0.7 mg/m^2 ^IV

**Week 25:**		Doxorubicin 30 mg/m^2 ^IV (+ 0.9% NaCl) Pre-med with chlorphenamine + metoclopramide

BLOODS:

**Haematology: **prior to each dose

Urine:- Dipstick after every cyclophosphamide dose

- Full urinalysis after week 7

*CARDIAC SCAN:*

At time of: - First doxorubicin dose

- Last doxorubicin dose

- 3 months post protocol completion

REPEAT VISITS:

If in complete remission, checks should be made at months 1, 2, 3 and 6

**NOTES:**

• For dogs < 10 Kg, use a 1 mg/Kg dose of doxorubicin.

• All treatment is discontinued after week 25 if in complete remission.

• If neutrophil count is < 3 × 10^9^/L wait 5-7 days and repeat haematology before giving chemo.

**• **If sterile haemorrhagic cystitis occurs on cyclophosphamide, discontinue and substitute **Chlorambuci**l (1.4 mg/kg PO) for subsequently scheduled cyclophosphamide injections.

**• **You can administer **L-asparaginase **(400 IU/kg IM) with each vincristine injection until CR is achieved.

**Table 5:** MOPP lymphoma protocol

DAY	DRUG	DOSE
0	Mechlorethamine	3.0 mg/m^2^
	Vincristine	0.75 mg/m^2^
	Procarbazine	50 mg/m^2^PO SIDx 14 days
	Prednisone	30 mg/m^2^PO SID × 14 days

7	Mechlorethamine	3.0 mg/m^2^
	Vincristine	0.75 mg/m^2^

Repeat on day 28, complete blood cell count on days 0, 7, 28 etc.

**Table 6:** Characteristics and outcome of canine patients treated with pump inhibitors and chemotherapy

PATIENT	AGE	TUMOR	PREV. TREATMENT	THERAPY	OUTCOME (MONTHS)
GREAT DANE	10	SPLENIC HSA	SURGERY METRONOMIC	METRONOMIC	CR 3

WHWT	8	LSA	MADISON MOPP	MOPP	CR 12

BULL MAST.	11	ALL	MADISON MOPP	MOPP	CR 3

WHWT	8	LSA	MADISON MOPP	MOPP	CR 8

ROTTW.	6	LSA	MADISON MOPP	MOPP	CR 3

BOXER	8	LSA	MADISON MOPP	MOPP	CR 7

BOXER	9	LSA	MADISON MOPP	MOPP	CR 5

DOGO	10	LSA	MADISON MOPP	MOPP	PR 3

WHWT	10	LSA	MADISON MOPP	MOPP	PD

BOXER	10	LSA	COP MOPP	MOPP	CR5+

LABRADOR	8	LSA	COP MOPP	MOPP	CR5+

BULL DOG	10	SKIN LSA	MADISON MOPP	MITOXANTRONE	CR5+

POODLE	12	LSA	COP MOPP	MOPP	DISCONTINUED

SETTER	14	ORAL SCC	MITOXANTRONE	MITOXANTRONE	DISCONTINUED

MIXED	11	NASAL CA	MITOXANTRONE	MITOXANTRONE	PR 3+

MIXED	10	OSA	BIOPSY	CLODRONATE	PR 7

MIXED	11	OSA	BIOPSY	CLODRONATE	PR 11

SCHNAUTZER	9	OSA	BIOPSY	CLODRONATE	DISCONTINUED

MIXED	15	TCC BLADDER	PIROXICAM	PIROXICAM	SD 7+

BEAGLE	12	TCC BLADDER	SURGERY MITOXANTRONE	MITOXANTRONE	CR 2+

ROTTWEILER	5	MELANOMA	SURGERY CARBOPLATIN	CARBOPLATIN	NED 5+

MIXED	10	MAMMARY CA	SURGERY MITOXANTRONE	MITOXANTRONE	NED 5+

MIXED	10	MAMMARY CA	CARBOPLATIN DOXORUBICIN	MITOXANTRONE	PR 9+

MIXED	11	FSA	SURGERY, ECT	MITOXANTRONE	NED 3+

MIXED	12	MAMMARY CA	SURGERY MITOXANTRONE	MITOXANTRONE	NED 4+

GERMAN	8	MAMMARY	SURGERY	MITOXANTRONE	NED 4+

SHEPHERD		CA	MITOXANTRONE		
HUSKY	13	MAMMARY	SURGERY	MITOXANTRONE	NED 5+
		CA.SA	MITOXANTRONE		

**Abbreviations: **ALL: acute lymphocytic leukemia; CA: carcinoma; CA.SA: carcinosarcoma; COP: cyclophosphamide, oncovin (vincristine), prednisone; ECT: electrochemotherapy; FSA: fibrosarcoma; HSA: hemangiosarcoma; LSA: lymphosarcoma; MOPP: mechlorethamine, oncovin (vincristine), prednisone, procarbazine; OSA: osteosarcoma; SCC: squamous cell carcinoma, WHWT: west highland white terrier. +: still alive.

Drugs schedule: doxorubicin 30 mg/m^2 ^every 21 days (pending hematological, cardiac and renal evaluation) up to 6 doses, carboplatin 300 mg/m^2 ^every 21 days, mitoxantrone 6 mg/m^2 ^every 21 days. For Madison and MOPP protocols see table 4 and 5

CONTROL COHORT: ten dogs were treated just with conventional chemotherapy during the study and their characteristics and therapies are summarized in table 7. There were 3 mixed breed dogs and one each of the following breeds: Setter, Boxer, Labrador, German Shepherd, Argentine Dogo, West Higland White Terrier and Rottweiler. Overall there were 5 lymphoma patients, one with acute lymphocytic leukemia, one with cutaneous carcinoma, one with liposarcoma and two affected by mammary carcinoma. All failed their therapies at different times but the owners elected to pursue additional chemotherapies without chemosensitizers.

**Table 7:** Characteristics and outcome of canine patients treated with chemotherapy alone for refractory cancers

PATIENT	AGE	TUMOR	**PREV**. TREATMENT	THERAPY	OUTCOME (MONTHS)
MIXED	11	LSA	MADISON + MOPP	MADISON	PD 1

SETTER	6	ALL	MADISON + MOPP	MADISON	PD 1

BOXER	9	SKIN CARCINOMA	DOXORUBICIN CARBOPLATIN	CARBOPLATIN	PR 2

WHWT	8	LSA	MADISON + MOPP	MADISON	PR 1

ROTTW	7	LSA	MADISON + MOPP	MADISON	PR 3

GERMAN	9	LSA	MADISON + MOPP	MADISON	PD 1

SHEPHERD			MOPP		

LABRADOR	9	LSA	MADISON + MOPP	MOPP	PD 2

MIXED	10	MAMMARY CARCINOMA	DOXORUBICIN CARBOPLATIN	DOXORUBICIN	PR 1

DOGO	10	LIPOSARCOMA	DOXORUBICIN CARBOPLATIN	CARBOPLATIN	PD 1

MIXED	10	MAMMARY CARCINOMA	DOXORUBICIN CARBOPLATIN	CARBOPLATIN	PR 2

**Abbreviations: **ALL: acute lymphocytic leukemia; LSA: lymphosarcoma; MOPP: mechlorethamine, oncovin (vincristine), prednisone, procarbazine; WHWT: west highland white terrier.

Drugs schedule: doxorubicin 30 mg/m^2 ^every 21 days (pending hematological, cardiac and renal evaluation) up to 6 doses, carboplatin 210 mg/m^2 ^every 21 days, mitoxantrone 6 mg/m^2 ^every 21 days. For Madison and MOPP protocols see table 4 and 5.

TOXICITY: Twenty-three dogs tolerated the dose of lansoprazole without need of reduction or discontinuation. Four dogs experienced grade 1 and two dogs had grade 2 gastrointestinal toxicity (diarrhea) that was managed with probiotics. Five dogs had flatulence (grade 1-3). One dog had grade 3 vomiting that required lansoprazole dose reduction (from 5 to 3 mg/kg). Three dogs had to be withdrawn due to lansoprazole intolerance. Hematological toxicity of chemotherapy was not worsened by the addition of PPI, being limited to two episodes of grade 1 neutropenia and one case of grade 2 neutropenia that did not require treatment. Seven out of ten controls experienced progressive anemia and neutrophilia with left shift due to poor responses to therapy or progressive disease.

**Figure 1:**
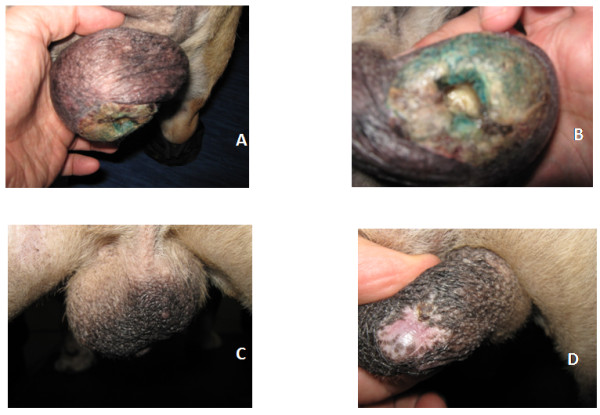
**Necrotized scrotal ulcer secondary to lymphosarcoma infiltration in a bull dog at presentation (A & B) and after initiation of rescue protocol (C & D)**.

EFFICACY: In terms of efficacy nineteen out of twenty-eight dogs experienced various degrees of response lasting from two to twelve months, these responses were more evident in dogs affected by lymphoma (9 out 11). A paradigmatic example is shown in Figure 1, depicting the complete regression of a necrotic lymphomatous ulcer upon addition of PPI to the chemotherapy regimen of a dog with refractory lymphoma. In osteosarcoma patients the efficacy consisted in potentiation of clodronate efficacy in dogs with progressive disease that resulted in 2 partial responses. An additional benefit, beside the arrest of tumor progression has been a significant improvement of their Karnofsky performance status, characterized by a significant decrease of the pain perception shown by a better weight bearing condition and weight gain. In dogs with melanoma, carcinoma and sarcoma the addition of lansoprazole resulted in high response rates as shown by table 6 with one melanoma patient that died of thoracic lymphosarcoma while still in remission for the melanocytic tumor.

Besides the osteosarcoma patients, two other dogs with advanced disease (one affected by acute lymphocytic leukemia and one by lymphoma) with a poor Karnofsky performance status, upon clinical response, moved to a lower grade condition (one moved from grade 3 to grade 1, the other from grade 2 to grade 1). On the contrary, only two dogs in the control group benefited from the continuation of chemotherapy, experiencing short lived partial responses (complete responses were not documented) while the others had progressive disease that ultimately resulted in their death. Upon questioning, all the owners reported a decreased/poor quality of life for their pets.

### Cats

PPI COHORT: Seven cats entered the study during the enrollment period, their characteristics and treatment schedules are summarized in table 8. All of them where Domestic Short Hair cats, four were females and three were males, age ranged from 7 to 12 years. Tumor types included three oral squamous cell carcinomas, two lymphomas, one fibrosarcoma and one breast carcinoma that failed chemotherapy or various combinations of surgery and chemotherapy. Upon recurrence or progressive disease, PPI inhibitor lansoprazole has been added to the chemotherapy protocol.

**Table 8:** Characteristics and outcome of feline patients treated with pump inhibitors and chemotherapy

PATIENT	AGE	TUMOR	**PREV**. TREATMENT	THERAPY	OUTCOME (MONTHS)
DSH	10	BREAST CARCINOMA	SURGERY MITOXANTRONE PD	MITOXANTRONE	PR 4

DSH	12	ORAL SCC	MITOXANTRONE PD	MITOXANTRONE	SD 2

DSH	10	ORAL SCC	SURGERY MITOXANTRONE PD	MITOXANTRONE	CR 5+

DSH	12	ORAL SCC	BIOPSY MITOXANTRONE PD	MITOXANTRONE	SD 6

DSH	12	LSA	MOPP × 3 months	MOPP	CR 6+

DSH	8	NASAL LSA	MOPP PD	MOPP	PR 3+

DSH	7	FSA	SURGERY, ECT × 2, CARBOPLATIN × 2 MITOXANTRONE × 2	MITOXANTRONE	PD

**Abbreviations: **DSH: domestic short hair; ECT: electrochemotherapy; FSA: fibrosarcoma; LSA: lymphosarcoma; MOPP: mechlorethamine, oncovin (vincristine), prednisone, procarbazine; SCC: squamous cell carcinoma. +: still alive.

Drugs schedule: doxorubicin 30 mg/m^2 ^every 21 days (pending hematological, cardiac and renal evaluation) up to 6 doses, carboplatin 210 mg/m^2 ^every 21 days, mitoxantrone 6 mg/m^2 ^every 21 days. For MOPP protocol see table 4 and 5.

CONTROL COHORT: Seven cats entered the study during the enrollment period, their characteristics are summarized in table 9. All but a Norwegian cat where Domestic Short Hair cats, three were females and four were males, age ranged from 5 to 13 years. All the owners of these patients elected the continuation of chemotherapy despite its declining efficacy.

**Table 9:** Characteristics and outcome of feline patients treated chemotherapy alone for chemoresistant cancers

PATIENT	AGE	TUMOR	**PREV**. TREATMENT	THERAPY	OUTCOME (MONTHS)
DSH	10	BREAST CARCINOMA	DOXORUBICIN CARBOPLATIN	CARBOPLATIN	PD 1

DSH	12	FSA	CARBOPLATIN	CARBOPLATIN	PR 2

DSH	10	ORAL SCC	MITOXANTRONE CARBOPLATIN	MITOXANTRONE	PD 1

DSH	12	ORAL FSA	DOXORUBICIN CARBOPLATIN	CARBOPLATIN	PD 1

DSH	12	LSA	MADISON MOPP	MOPP	PR 2

DSH	7	FSA	SURGERY, DOXORUBICIN CARBOPLATIN	CARBOPLATIN	PD 1

NORVEGIAN	7	LSA	MADISON MOPP	MOPP	PD 2

**Abbreviations: **DSH: domestic short hair; FSA: fibrosarcoma; LSA: lymphosarcoma; MOPP: mechlorethamine, oncovin (vincristine), prednisone, procarbazine; SCC: squamous cell carcinoma. +: still alive.

Drugs schedule: doxorubicin 30 mg/m^2 ^every 21 days (pending hematological, cardiac and renal evaluation) up to 6 doses, carboplatin 210 mg/m^2 ^every 21 days, mitoxantrone 6 mg/m^2 ^every 21 days. For Madison and MOPP protocols see table 4 and 5.

TOXICITY: Regarding the cats, gastrointestinal side effects were limited to mild anorexia in one cat while they were not noted in the remaining six. Systemic toxycoses were limited to one cat that experienced a severe neutropenia (grade 3) leading to infection (grade 3) that required hospitalization and antibiotic therapy. Again, the control group patients had a much worse tolerance to the therapy due to progressive disease.

**Figure 2:**
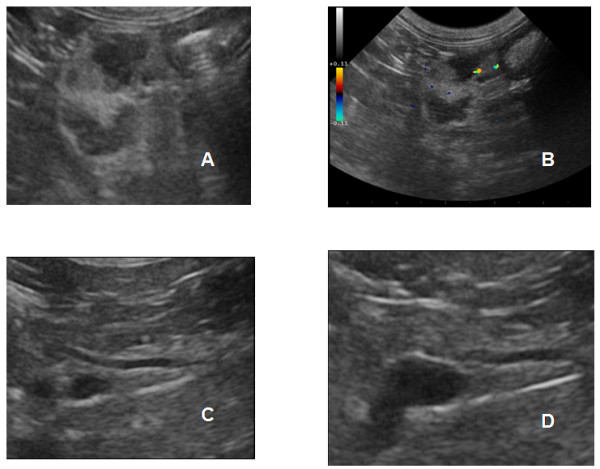
**Lymphosarcoma nodules in the pancreas of a cat, diagnosed by fine needle aspiration with ultrasonographic guidance**. Ultrasonographic appearance of pancreatic lesions at presentation (A & B) and after initiation of rescue protocol (C & D).

EFFICACY: Six cats benefited with variable degrees of response from the therapy. Four cats showed tumor response (2 CR, 2 PR ), two cats had stabilization of the disease and one was a non responder. The complete responders were a cat with an aggressive lymphoma that had spread to the pancreas, the other was a cat with oral squamous cell carcinoma. Figure 2 shows the regression of the pancreatic dissemination in the lymphoma patients. The partial responders were a cat with nasal lymphoma and a cat with mammary carcinoma. Interestingly, the two cats with oral squamous cell carcinoma that had stabilization of their disease showed a significant improvement of their quality of life, in terms of decreased bleeding, activity level, interaction with the owners, improved grooming and increased appetite. In terms of performance, they moved from a grade 3 to a grade 1 Karnofsky status, mostly due to a significantly decreased tumor induced pain. Again, the response rate in the control group was much smaller with only two control cats (one with a soft tissue sarcoma and the other with lymphoma) experiencing a brief partial response lasting two months during the therapy (see Table 9).

At the time of writing a total of 15 patients (12 dogs and 3 cats) are still alive and periodically monitored leading to a survival rate of 44.4% and 42.8%, respectively. Questioning the owners regarding their degree of satisfaction with the outcome of the therapy yielded a total of 85% of appreciation that ranged from "moderate" to "enthusiastic". The major cause of complain being the gastrointestinal side effects (specifically the flatulence) experienced by some dogs rather than the degree of tumor response.

## Discussion

From a strictly chemical evaluation of proton pump inhibitors, it is conceivable that being pro-drugs needing acidity to be transformed in the active drug [39], they might be specifically active in the acidic tumor environments. Some reports inferred that metastatic tumors are more acidic then primary tumors, but also that solid tumors, either carcinoma or melanomas or sarcomas, are more acidic than systemic tumors (i.e. leukemia) [40]. Therefore, it can be speculated that proton pump inhibitors might be more active against very malignant, often entirely unresponsive to current therapies, tumors. These observations are at least partially contradicted by our findings since in both PPI cohorts we observed extended responses ( > 6 months) in patients affected by solid tumors as well as in patients with hematological malignancies. In particular we found intriguing the consistent responses obtained both in canine and feline lymphoma patients that had shown a significant refractoriness to standard multi-drug protocols. Pooling together dogs and cats with lymphoma 8 patients out of 14 (57%) had responses in excess of 5 months. When examining the outcome of patients with advanced solid tumors, we observed a broad spectrum of tumors vulnerable to our novel strategy. Among them, two of our canine responders suffered neoplasms that are usually refractory to current treatments, including a spinal osteosarcoma and a metastatic anal sac carcinoma. Sarcomas have been shown to be clearly acidic, also by their ability to capture Acridine Orange [41-43]. However, also metastatic neoplasms are particularly acidic as has been shown for human melanomas, that thrive in particularly acidic microenvironments, as compared to primary tumors [27]. In our study, the patient affected by melanoma, showed responsiveness for the treatment of gross disease, being consistent with the observation that acidic condition increases susceptibility of metastatic melanoma cells to proton pump inhibitors [27]. We have showed a dramatic decrease of pain in dogs with osteosarcoma and in cats affected by oral squamous cell carcinoma invading the maxilla or the mandible, following clinical response to the treatment with lansoprazole. This stabilization of tumor mass led to palliation of bone cancer pain, resulting in improved quality of life as shown by increased activity levels, improved grooming and feeding ability, and weight gain, as well. Modulation of tumor microenvironment pH may be postulated for pain control as well [44], but it is conceivable that a general acidosis is involved in advanced cancer, contributing to the establishment of the typical cachexia of cancer patients [45]. These data are further substantiated by comparing the quality of life of our PPI cohorts with that of controls that showed progressive weight loss, decreased appetite, lower tolerance to chemotherapy (as shown by worse hematological and gastrointestinal toxicities) and the general tendency to move toward higher Karnowski levels. Bone cancer induces bone lysis and remodeling leading to mechanical bone deformation and inducing local tissue acidosis, as well, which in turn may activate pain receptors through several molecular mechanisms, particularly by activation of the capsaicin receptor (transient receptor potential vanilloid, TRPV1) [46,47]. Compelling evidence has shown that TRPV1 is a crucial signal molecule in the development of physiological and pathological pain [46]. In fact, it has been shown that the reduction of pH may induce both osteoclast-mediated bone reabsorption, with direct stimulation of nociceptive receptors, and the switch-on signal transmission by acid-sensing channels such as TRPV 1 located on pain-sensing neurons [46-48]. The high response rate observed in this study is extremely promising, considering that most patients suffered advanced cancers refractory to chemotherapy. This PPI-induced response to anti-tumor therapy provides the proof of concept that inhibition of the proton pump may represent a new approach in the struggle against cancer. The mechanisms of this efficacy lay in both the improvement of chemotherapy by countering the acid mileu of the tumors [26], but also the induction of tumor self-digestion triggered by the increased cytoplasmic protonation [27-31].

## Conclusions

The results obtained in pets with spontaneous neoplasms will be instrumental for the planning of further investigations to be pursued in humans, thus hopefully shortening the time frame necessary for the adoption of this approach in clinical oncology [49,50]. Our data are particularly comforting for patients with hematological malignancies, due to the relatively high number of enrolled canine and feline patients. The data on solid tumors response, albeit promising need to be further substantiated by the enrollment of other patients. Studies are currently ongoing for several specific solid tumor histotypes that will be instrumental for the standardization of the PPI protocols.

Extending the number of patients treated with this new approach is needed to further support the result of this study and to identify both the more sensitive tumor histotypes and the better PPI and conventional chemotherapy combinations. Prospective studies will be conducted to evaluate the efficacy of PPI administered to non-chemoresistant tumors. Finally, we want to emphasize that this study adds much on the set up of new anti-tumor therapies based on drugs with low cost, minimal toxicity, effective pain palliation and antitumor efficacy.

## List of abbreviations

PPI: proton pump inhibitors; TRPV1: transient receptor potential vanilloid.

## Competing interests

The authors declare that they have no competing interests.

## Authors' contributions

EPS, GC and SF conceived the study and participated in its design and coordination and wrote the article; MdB G, SB, FC, GB, IP and FM helped with the clinical management of the patients, AB participated in the design of the study and performed the histopathological analysis. All the authors read and approved the final manuscript.
